# Induced Abortion

**Published:** 1883-06

**Authors:** 


### INDUCED ABORTION.

A recent writer has made the statement that over six thousand women die annually in the United States from attempts at induced abortion.—*George O. Woody, M.D., in Med. and Surg. Reporter.* Large as this number is, we would be glad indeed to be assured that it is really no greater—one in a little more than every sixteen hundred of the ten million of women in the Union.

But this social evil, hideous as its prevalence and immorality are, is a bagatelle compared with the enormity of *partial sexual intercourse and other devices to prevent conception.* One of the most important articles of a fashionable woman's trousseau now is a *syringe.*

No wonder it is told of a distinguished gynecologist of this city that on one occasion when annoyed beyond endurance by the conversation of a fashionable woman whose love of pleasure was stronger than her principles, and greater than her affection for children, he passionately exclaimed: "My God, madam, hell is full of such women."

It was, not long since, the remark of one of the best known and most popular physicians of Georgia that there is one fact in relation to this subject with which he is often in painful contact that astonishes him beyond measure, viz: the large and increasing number of refined, intelligent, and apparently Christian women, who show a most child-like innocence of all conception of any immorality in such a practice. Yet these Southern ladies are amply supplied with Bibles in their homes, hear the gospel preached every Sabbath, and were trained by mothers to whom a suggestion of the kind would have been an unspeakable horror.

Where is the author of Flora McFlimsy? O, for the lash of some modern Juvenal!

